# Investigation of prescribing behavior at outpatient settings of governmental hospitals in eastern Ethiopia: an overall evaluation beyond World Health Organization core prescribing indicators

**DOI:** 10.1186/s40545-018-0152-z

**Published:** 2018-10-15

**Authors:** Tigist Gashaw, Mekonnen Sisay, Getnet Mengistu, Firehiwot Amare

**Affiliations:** 1 Department of Pharmacology and Toxicology, School of Pharmacy, College of Health and Medical Sciences, Haramaya University, P.O. Box 235, Harar, Ethiopia; 2 Department of Pharmacy, College of Medicine and Health Sciences, Wollo University, Dessie, Ethiopia; 3 Department of Pharmacy Practice, School of Pharmacy, College of Health and Medical Sciences, Haramaya University, P.O. Box 235, Harar, Ethiopia

**Keywords:** Prescribing behavior, WHO, Prescribing indicators, Eastern Ethiopia

## Abstract

**Background:**

Rational prescribing remains an important component of rational drug use. The World Health Organization (WHO) standardized and validated core prescribing indicators for evaluating prescribing pattern of drugs. The prescribing practice has been shown to deviate from national and WHO guidelines in Ethiopia. The aim of this study was; therefore, to investigate the overall prescribing behavior of four governmental hospitals: Hiwot Fana Specialized University Hospital (HFSUH), Federal Harar Police Hospital (FHPH), Jugel Hospital (JH) and Southeast Command III Hospital (SECIIIH), Harar, eastern Ethiopia.

**Methods:**

Hospital based retrospective cross-sectional study was employed to evaluate outpatient prescriptions dispensed from January 1 – December 31, 2016. A total of 2400 prescriptions (600 from each hospital) were assessed. A combination of prescription completeness and prescribing indicator forms were used to collect the data.

**Result:**

From a total of 2400 prescriptions reviewed, only HFSUH and FHPH were using standard prescription at prevalence of 92.5 and 99.8%, respectively. Name and weight of the patient were the most and the least commonly recorded information, respectively. A total of 5217 drugs were prescribed with an average number of drugs per encounter to be 2.17 (±0.39) and the highest value (2.60) was observed at FHPH. The frequency of administration was the most commonly written component (85.0%) with an average of 1.85 per prescription. Among all prescriptions analyzed, the percentage of encounters with antimicrobials and injectables prescribed were 66.9 and 26.5%, respectively. The prevalence of drugs prescribed with generic name and from essential drug list were 4644 (89.01%) and 4613 (88.42%), respectively. Among health professional related information, dispenser name was the least documented in all hospitals with the prevalence being 3.9%.

**Conclusion:**

JH and SECIIIH were not using standard prescriptions at all during the review period. Besides, some important components of the prescription such as age, sex and diagnosis were not properly recorded or missed at all in the selected hospitals. The tendency of prescribing drugs with dose and dosage form was very poor. Overall, none of the core prescribing indicators was in line with the WHO standards. These and other related problems should be investigated in-depth to find out the underlying problems for which interventional strategies can be designed to reverse this worrying practice.

## Background

Medicines play a crucial role in the delivery of healthcare service across the globe. Nevertheless, not only is the availability of medicines important, but also rational prescribing remains an important component of treating patients as this may be a reflection of the quality of care delivered to them and the community at large [1, 2]. Appropriate use of medicines plays a pivotal role in reducing global morbidity and mortality. However, the World Health Organization (WHO) has reported that around 50% of all medicines are prescribed, dispensed or sold inappropriately and more than half of all patients fail to take their medicines as prescribed or dispensed. Such inappropriate use is wasteful of resources and causes patient harm in terms of lack of satisfactory outcome, serious adverse events and increased antimicrobial resistance (AMR) [2, 3]. To minimize these problems, WHO developed and validated core drug use indicators for prescribing, patient care and facility specific studies. The prescribing indicators measure the performance of prescribers in five key areas (average number of drugs per prescription, percentage of drugs prescribed by generic name, percentage of prescriptions containing antibiotics, percentage of prescriptions containing injectable drugs, and percentage of drugs prescribed from the latest edition of national Essential Drug Lists (EDL) or formulary) [4]. Even if several studies have been conducted on the area of prescribing practice with the aid of such five core prescribing indicators in various parts of Ethiopia [5–10], none of these findings holistically addressed the overall features of prescription beyond assessing prescribing practice with the usual indicator studies. This study attempted to combine the prescription features and completeness with WHO prescribing indicator forms to generate a cumulative body of evidence for decision making. Prescribing indicators are useful for investigating major problem areas of prescribing practice in quantitative basis. These core indicators, however, failed to address the nature of the prescription, its standards, and presence of essential components (patient, drug and health professional related details), among others [4]. Moreover, a study of prescribing behavior is an important tool to determine rational drug therapy and maximize utilization of resources [9]. Drug utilization reports that assess drug consumption and prescribing patterns should be done on regular basis for monitoring and evaluation of drug use and national drug policy at large [11]. So, this study aimed to investigate the prescribing behavior in outpatient settings of four government hospitals of Harar town, Eastern Ethiopia.

## Methods

### Study area, design and period

Harar is located 526 km away from the capital of Ethiopia, Addis Ababa to the East. Harari region is one of the nine national regional states of Ethiopia, with the town of Harar as its capital. Harar is one of the most popular historical towns in the eastern part of Ethiopia. The town has a projected total population of 203,438 with male to female ratio of 1.01:1 in 2010. Harar town is divided in to 19 kebeles. In the town, there are four governmental hospitals, two private hospitals, and eight health centers [12]. The study was conducted in four governmental hospitals: Hiwot Fana Specialized University Hospital (HFSUH), Jugel Hospital (JH), Federal Harar Police Hospital (FHPH) and South-East command III Hospital (SECIIIH). HFSUH is a tertiary care teaching hospital of Haramaya university and hosts majority of patient attendees per day from the Harar town and nearby areas. JH is a regional hospital of Harari regional state. Whereas, FHPH and SECIIIH are special government hospitals in which the service is primarily delivered to police and military clients and their relatives. Hospital based retrospective cross-sectional study was employed to investigate the overall prescribing behavior of the year 2016 in the above-mentioned hospitals from July 1 to September 30, 2017.

### Population and inclusion/exclusion criteria

All out-patient prescriptions dispensed from January 1 to December 31, 2016 from each hospital were taken as study populations. However, prescriptions that contained only medical supplies like glove, and syringe; those prescriptions contained drug which were found illegible and prescriptions which were brought from outside of the selected hospitals were excluded from the study.

### Sample size determination and sampling technique

Based on the current WHO criteria [4], 600 prescriptions were taken from each hospital (a total of 2400 prescriptions) to assess completeness of the prescription and prescribing pattern in the hospitals. A systematic random sampling technique was employed to collect 600 eligible prescriptions from each hospital once the annual prescriptions had been arranged in the chronological order. The sampling interval was 150, 119, 28 and 43 for HFSUH, JH, FHPH and SECIIIH, respectively.

### Data collection tools and methods

Data were collected by using data collection tool (observational checklist) containing two components: 1) prescription completeness assessment form customized from manuals developed and validated by Food, Medicine and Healthcare Administration and Control Authority (FMHACA) of Ethiopia and WHO [13, 14]. The manuals emphasized that prescriptions should be standardized (the paper should have the name of the local health facility, contact details and serial numbers (if any) as well as all essential components of the prescription in preprinted form). In addition, the prescription should be considered complete if it contains patient, drug and prescriber and dispenser related directions prepared in preprinted form and with sufficient space for hand filling. 2) prescribing indicator form developed and validated by WHO for assessing prescribing practice at outpatient settings of health facilities [4]. The prescribing indicators include average number of drugs per prescription, percentage of drugs prescribed by generic name, percentage of prescribing encounters containing antibiotics, percentage of prescribing encounters containing injectable drugs, and percentage of drugs prescribed from the latest edition of national EDL or formulary. Data were collected retrospectively from individual prescription and filled in structured observational check list accordingly.

### Data quality control

To ensure the data quality, data collectors (pharmacy technicians) and supervisors (pharmacists) were trained by the principal investigators for 3 days. The data collection tool was pretested at HFSUH (data not included in the actual study and solely used for amendment) and necessary modifications were undertaken in the tool. The principal investigators and supervisors underwent frequent checks on the data collection process to ensure the completeness and consistency of the collected data. Data were cleaned to remove inconsistencies and missing values on daily basis.

### Data analysis and presentation

Data were entered to EPI-Data Version 3.1 and exported to and analyzed with Statistical Package for Social Sciences version 20.0 (IBM statistics, Armonk, NY, United States). Univariate analysis was employed to summarize the findings. Prescribing indicators were computed using the formula adopted by the WHO for prescribing indicators assessment [4, 15]. Finally, the data were evaluated against WHO standards and presented in tabular way.

## Result

### Patient related information on a prescription

A total of 2400 prescriptions (600 from each hospital) were analyzed in the study. The analysis showed that standard prescriptions were used in HFSUH and FHPH at a prevalence of 555 (92.5%) and 599 (99.8%), respectively. Almost all prescriptions had a medical registration number in HFSUH (*n* = 598, 99.7%) and FHPH (*n* = 599, 99.8%) whereas lowest recording practice was observed in JH (*n* = 332, 55.3%). The overall prevalence of registration number in prescription was found to be 2082(86.8%). Moreover, the examination of patient related information on the prescription revealed that name of the patient was the most commonly recorded information (*n* = 2254, 93.9%) while weight and address of the patient were the least commonly recorded components obtained from 0.9 and 1.1% prescriptions, respectively (Table 1).

**Table 1:** Patient related information in prescriptions dispensed from Jan - Dec 2016 at four governmental hospitals of Harar town (*n* = 2400)

Patient related information	HFSUH (*n* = 600)	FHPH (*n* = 600)	JH (*n* = 600)	SECIIIH (*n* = 600)	Total (2400)
Use of standard prescription	555 (92.5)	599 (99.8)	0(0)	0(0)	1154(48.1)
Medical registration number	598 (99.7)	599 (99.8)	332(55.3)	553(92.2)	2082(86.8)
Date of prescription	367 (61.2)	497(82.8)	497(82.8)	590(98.3)	1951(81.3)
Name of patient	599 (99.8)	600 (100.0)	577(96.2)	478(79.7)	2254(93.9)
Age	527 (87.8)	117 (19.5)	343(57.2)	58(9.7)	1045(43.5)
Weight	18 (3.0)	3 (0.5)	2(0.3)	0(0)	23(0.9)
Sex	522 (87.0)	206 (34.4)	357(59.5)	32(5.3)	1117(46.5)
Patient Address	18 (3.0)	7 (1.2)	2(0.3)	0(0)	27(1.1)
Diagnosis	236 (39.3)	20 (3.3)	26(4.3)	0(0)	282(11.8)

*Abbreviations***:** *HFSUH* Hiwot Fana Specialized University Hospital, *FHPH* Federal Harar Police Hospital, *JH* Jugel Hospital, *SECIIIH* Southeast command III Hospital

### Number of drugs and drug related information on prescriptions

As shown in Table 2, a total of 5217 drugs were prescribed from 2400 prescriptions giving an average value of 2.17 (±0.39) drugs per prescription. The highest and the lowest number of drugs per encounter were observed at FHPH and HFSUH, respectively. Generally, large proportion of the prescriptions contained two drugs (*n* = 874, 36.4%) followed by a single drug (*n* = 733, 30.5%). Moreover, analysis of drug-regimen information indicated that the route and frequency of administration were the most commonly recorded components with a frequency of 4435 (85.0%) and 4270 (81.8%), respectively. In contrary, drugs prescribed with dose and dosage forms were found to be 1429 (27.4%) and 1708 (32.7%), respectively (Table 2). The mean value of components of drug regimen per encounter showed that the frequency of drug administration had the highest value (mean = 1.85) whereas the dose of the drug had a value of 0.59 (Table 3).

**Table 2:** Drug related information on prescriptions dispensed from Jan 1 - Dec 31, 2016 at four governmental hospitals of Harar town

Drug related information	Frequency (%)				
	HFSUH	FHPH	JH	SECIIIH	Overall
Drugs per prescription (*n* = prescription in each hospital)	(*n* = 600)	(*n* = 600)	(*n* = 600)	(*n* = 600)	(*n* = 2400)
One	309 (51.5)	129 (21.5)	139 (23.2)	156 (26.0)	733 (30.5)
Two	201 (33.5)	169 (28.2)	209 (34.8)	295 (49.2)	874 (36.4)
Three	64 (10.7)	177 (29.5)	170 (28.3)	129 (21.5)	540 (22.5)
Four	15 (2.5)	76 (12.7)	61 (10.2)	19 (3.2)	171 (7.1)
Five	9 (1.5)	39 (6.5)	17 (2.8)	1 (0.2)	66 (2.7)
Six and above	2 (0.4)	10 (1.7)	4 (0.7)	0 (0)	16 (0.6)
Average number of drugs per encounter	1.70	2.60	2.37	2.02	2.17 (± 0.39)
Drug regimen related information (*n* = drugs per hospital)	(*n* = 1021)	(*n* = 1560)	(*n* = 1422)	(*n* = 1214)	(*n* = 5217)
Dose	317 (31.04)	502 (32.18)	234 (16.5)	376 (30.9)	1429 (27.4)
Strength	661 (64.74)	1039 (66.60)	1161 (81.6)	1036 (85.3)	3897 (74.7)
Dosage Form	153 (14.99)	302 (19.36)	169 (11.9)	1084 (89.3)	1708 (32.7)
Route	816 (79.92)	1098 (70.38)	1208 (84.9)	1148 (94.6)	4270 (81.8)
Frequency	716 (70.13)	1414 (90.64)	1171 (82.3)	1134 (93.4)	4435 (85.0)
Duration	478 (46.82)	409 (26.21)	981 (68.9)	868 (71.5)	2736 (52.4)
Quantity	474 (46.43)	1087 (69.68)	515 (36.3)	1130 (93.1)	3206 (61.5)

*Abbreviations HFSUH* Hiwot Fana Specialized University Hospital, *FHPH* Federal Harar Police Hospital, *JH* Jugel Hospital, *SECIIIH* Southeast command III Hospital

**Table 3:** Mean-value of components of drug regimen per prescribing encounter dispensed from Jan 1-Dec 31, 2016

Components	Mean value				
	HFSUH (*n* = 600)	FHPH (*n* = 600)	JH (*n* = 600)	SECIIIH (*n* = 600)	Overall (*n* = 2400)
Dose	0.53	0.84	0.39	0.63	0.59
Strength	1.10	1.73	1.91	1.73	1.62
Dosage Form	0.26	0.50	0.28	1.81	0.71
Route	1.36	1.84	2.01	1.75	1.78
Frequency	1.19	2.36	1.95	1.89	1.85
Duration	0.80	0.84	1.64	1.45	1.14
Quantity	0.79	1.81	0.86	1.88	1.34

*Abbreviations: HFSUH* Hiwot Fana Specialized University Hospital, *FHPH* Federal Harar Police Hospital, *JH* Jugel Hospital, *SECIIIH* Southeast command III Hospital

### Distribution of antimicrobials and antibacterial agents per encounter

Among the 2400 prescriptions reviewed, the magnitude of antimicrobial (s) and antibacterial (s) containing prescriptions were 1605(66.9%) and 1473(61.4%), respectively. Almost half of the prescriptions contained a single antimicrobial and/or antibacterial agent. From the 5217 drugs prescribed, 2082 (39.9%) were antimicrobial agents from which 1671 (80.25%) were antibacterial agents and the average values per encounter were found to be 1.29(±0.03) and 1.13(±0.08) for antimicrobial and antibacterial agents, respectively (Table 4).

**Table 4:** Distribution of antimicrobials and antibacterial agents per encounter in prescriptions dispensed from Jan 1 - Dec 31 2016 at four governmental hospitals of Harar town

No. per encounter	HFSUH (*n* = 600)		FHPH (*n* = 600)		JH (*n* = 600)		SECIIIH (*n* = 600)		Overall (*n* = 2400)	
	AMA	ABA	AMA	ABA	AMA	ABA	AMA	ABA	AMA	ABA
One	267 (44.5)	292 (43.5)	300 (50.0)	319 (53.2)	274 (35.7)	303 (50.5)	390 (65.0)	374 (62.3)	1231 (51.29)	1288 (53.67)
Two	85 (14.2)	46 (7.7)	96 (16.0)	61 (10.2)	97 (16.2)	50 (8.3)	55 (9.2)	17 (2.8)	333 (13.87)	174 (7.25)
Three	10 (1.7)	1 (0.2)	11 (1.8)	3 (0.5)	13 (2.2)	6 (1)	1 (0.2)	0 (0.0)	35 (1.45)	10 (0.41)
Four	1 (0.2)	0 (0.0)	2 (0.3)	0 (0.0)	2 (0.3)	0 (0.0)	0 (0.0)	0 (0.0)	5	0
Five	0 (0.0)	0 (0.0)	1 (0.2)	1 (0.2)	0(0.0)	0 (0.0)	0 (0.0)	0 (0.0)	1	1
Total Rx^a^	363	339	410	384	386	359	446	391	1605 (66.9%)	1473 (61.4%)
Total drugs	471	387	538	455	515	421	558	408	2082	1671
Average drug per encounter	1.30	1.14	1.31	1.18	1.33	1.17	1.25	1.04	1.29 (±0.03)	1.13 (±0.08)

*Abbreviations AMA* antimicrobial agent, *ABA* antibacterial agent, *HFSUH* Hiwot Fana Specialized University Hospital, *FHPH* Federal Harar Police Hospital, *JH* Jugel Hospital, *SECIIIH* Southeast command III Hospital

^a^Prescriptions containing antimicrobial and/or antibacterial

### WHO core drug use indicators

The evaluation of prescribing practice using WHO core prescribing indicators revealed that the percentage of encounters with injection (s) and antimicrobial (s) prescribed were 636 (26.5%) and 1605 (66.9), respectively. The percentage of drugs prescribed with generic name and EDL of Ethiopia were 4644(89.01%) and 4613 (88.42%), respectively (Table 5).

**Table 5:** WHO core prescribing indicators in four governmental hospitals of Harar town from Jan 1 – Dec 31, 2016

WHO core prescribing indicators	HFSUH	FHPH	JH	SECIIIH	Total	WHO standard
Encounters with injection(s) prescribed (*n* = 600)	262 (43.67)	123 (20.5)	219 (36.5)	32 (5.3)	636 (26.5)	< 25% (13.4–24.1%)
Encounters with antimicrobial (s) prescribed (*n* = 600)	363 (60.5)	410 (68.33)	386 (64.33)	446 (74.3)	1605 (66.9).	< 30% ^a^(20.0–26.8%)
Encounters with antibacterial (s) prescribed (*n* = 600)	339 (56.5)	384 (64.0)	359 (59.8)	391 (65.2)	1473 (61.4)	–
Percentage of drugs with generic name (*n* = total drugs per hospital)	960 (94.02)	1350 (86.53)	1260 (88.60)	1074(88.46)	4644 (89.01)	100%
Percentage of drugs from EDL (*n* = total drugs per hospital)	960 (94.02)	1352 (86.67)	1103 (77.57)	1198 (98.68)	4613 (88.42)	100%

Abbreviations: *HFSUH* Hiwot Fana Specialized University Hospital, *FHPH* Federal Harar Police Hospital, *JH* Jugel Hospital, *SECIIIH* Southeast command III Hospital

^a^indicates the maximum acceptable value in tropical countries where infectious diseases are highly prevailing

### Health professional related information

From all prescriptions included in the study, 2133 (88.9%) prescriptions contained the signature of the prescriber and 1083(45.1%) contained the prescriber name. Dispenser related information was found very low with the prevalence being 94 (3.9%) and 152 (6.3%) prescriptions for dispensers’ name and signature, respectively (Table 6).

**Table 6:** Healthcare professional’s information in prescriptions dispensed from Jan 1 - Dec 31 2016 at four governmental hospitals of Harar town

Health professional related information	HFSUH (*n* = 600)	FHPH (*n* = 600)	JH (*n* = 600)	SECIIIH (*n* = 600)	Total (*n* = 2400)
Prescriber name	360 (60.0)	327 (54.5)	171(28.5)	225(37.5)	1083(45.1)
Prescriber signature	582 (97.0)	586 (97.7)	396(66.0)	569(94.8)	2133(88.9)
Dispenser name	86 (14.3)	0 (0.0)	1(0.2)	7(1.2)	94(3.9)
Dispensers signature	146 (24.3)	1 (0.2)	1(0.2)	4(0.7)	152(6.3)

*Abbreviations: HFSUH* Hiwot Fana Specialized University Hospital, *FHPH* Federal Harar Police Hospital, *JH* Jugel Hospital, *SECIIIH* Southeast command III Hospital

### Frequently prescribed drugs

From all drugs (*n* = 5217) prescribed, the most commonly prescribed classes of drugs were antimicrobial agents (*n* = 2036, 39.2%) followed by analgesics and anti-inflammatory agents (*n* = 1548, 29.67%) and gastrointestinal agents (*n* = 555, 10.64%). Looking at the individual drugs, amoxicillin (*n* = 497, 24.41%), diclofenac (*n* = 594, 38.37) and omeprazole (*n* = 206, 37.11%) were the top prescribed drugs under antimicrobials, analgesics and anti-inflammatory drugs and gastrointestinal agents, respectively (Table 7).

**Table 7:** Common pharmacological classes of drugs prescribed and top three individual drugs per class in prescriptions dispensed from Jan 1-Dec 31, 2016

Top drug classes	N (%)	Top three drugs per class	N (%)
Antimicrobial agents	2036 (39.02)	Amoxicillin	497 (24.41)
		Ciprofloxacin	276 (13.56)
		Metronidazole	202 (9.92)
Analgesics	1548 (29.67)	Diclofenac	594 (38.37)
		Paracetamol	388 (25.06)
		Tramadol	239 (15.44)
Gastrointestinal drugs	555 (10.64)	Omeprazole	206 (37.11)
		Cimetidine	115 (20.72)
		Plasil	104 (18.74)
Cardiovascular drugs	260 (4.98)	Furosemide	62 (23.85)
		Nifedipine	56 (21.54)
		Spironolactone	26 (10.0)
Vitamins & Minerals	253 (4.85)	Multivitamin	84 (33.20)
		Pyridoxine	28 (11.06)
		Vitamin B complex	21 (8.30)
Antihistamines	200 (3.83)	Chlorpheniramine	104 (52.0)
		Almetamin	52 (26.0)
		Diphenhydramine	10 (5.0)
Respiratory drugs	122 (2.33)	Salbutamol	58 (47.54)
		Cough syrup	49 (40.16)
		Theophydrine	9 (7.38)
Antidiabetic agents	119 (2.28)	Glibenclamide	45 (37.82)
		Metformin	40 (33.61)
		Insulin	25 (21.0)
Central nervous system drugs	86 (1.65)	Amitriptyline	33 (38.37)
		Fluoxetine	17 (19.77)
		Chlorpromazine	14 (16.28)
Others	38 (0.73)		
Total drugs	5217 (100)		

## Discussion

In this study, a total of 2400 prescribing encounters were reviewed from four government hospitals (600 prescriptions for each hospital). Compared to previous studies, this study tried to holistically address the prescribing behavior with better sample size and annual coverage of prescriptions in the four government hospitals of Harar town. In the review process, the general feature of prescriptions and its completeness was assessed. The prescribing practice was evaluated against the WHO standard [4]. Rational prescribing can have significant impact on the drug utilization process. Prescribers have become using substandard prescription papers and have also been practicing the standard format in the wrong way. Missing the drug regimens including the dosage, and duration of therapy might have contributed for emergence of drug resistance, toxicological issues, and poor treatment outcomes, among others [16]. Such baseline survey can be taken as a good input for designing and implementation of interventional studies. Most interventions undertaken to combat inappropriate use of medicines have been educational in nature, have had a relatively small impact and have not taken into account the determinants of behavior. A combination of interventions, involving managerial as well as educational components, appears to be more effective than a single intervention [3].

Regarding the general feature of prescription and its completeness, only HFSUH and FHPH were using standard prescriptions with nearly 100% practice observed in FHPH. However, JH and SECIIIH didn’t use standard prescriptions at all. Instead, they were using a piece of paper printed somewhere else and without containing essential components in the preprinted form. Such act of negligence observed throughout the review period is a good indication of how much irrational prescribing has becoming a huge concern. Almost all prescriptions had a medical registration number in HFSUH and FHPH whereas lowest recording practice was observed in JH. It is imperative that all prescription papers, irrespective of its content and form, would have contained the name of a patient to whom the drug was prescribed. Oddly enough, patient name was recorded in 79.7% prescription indicating the absence of this essential component in one out of five prescriptions in SECIIIH. Missing the name of the patient might create a room for the dispensers to issue the drug (s) without adequate counseling and even to the wrong patient. Even if the importance is not that much profound, the overall recording practice of the weight of patients was less than 1%. Considering drug therapy in pediatric patients, age and weight are among the essential components for ease of calculating dosage and correcting regimen errors [17, 18]. Nearly one-tenth of prescriptions contained diagnosis with the lowest (zero) value observed in SECIIIH. Recording diagnosis not only shows the transparency and confidence of the prescriber but also guides the dispenser and the patients about the disease condition being treated. Moreover, such poor recording practice might open a way for extravagant and overprescribing without justifiable reason [13, 14]. This and related gaps might create loss of trust and confidence among healthcare providers and patients in the healthcare system at large.

In contrary to our findings, Alkot et al. reported better results indicating the presence of name and age of the patients in all prescriptions and omission of diagnosis in only 1.56% of prescriptions [18]. In concordant with our findings, Admassie et al. indicated that the address of the patient and diagnosis were omitted in 97.29 and 99.99% prescriptions, respectively. Besides, patients’ age, sex and card number were only written in 86.64, 67.93 and 73.54% prescriptions, respectively [10].

Coming to drug related information, 5217 drugs were prescribed from all prescribing encounters reviewed. The average number of drugs per encounter was found to be 2.17(±0.39). Generally, larger proportion of prescription contained two drugs. Looking at the components of drug regimen, the most and least commonly recorded components were frequency and dose, respectively. Similarly, the mean value of frequency and dose per prescribing encounter were found to be 1.85 and 0.59, respectively. Almost similar behavior was observed across hospitals in recording the dose of medications. Even if dose should have been written in simple terms such as one tablet, two capsules, two teaspoons and the like, we had observed a significant mix up of strengths and doses and/or omitting or replacing doses with the strength of drugs. This might be partly ascribed to act of negligence or else lack of trainings on good prescribing practice. Overall, route of administration was the second most commonly recorded component of drug regimen. Nearly half of the drugs were prescribed without specified duration with poorest practice seen in FHPH. However, this hospital compensated such gap by having better record of total quantity of drugs for ease dispensing and inspection. In contrary to our finding, Alkot et al. reported that the dose and duration of therapy were missed only in 1.01 and 14.9% prescriptions, respectively [18]. Missing the duration of therapy might have resulted profound health realted problems including AMR, treatment failure and toxicological issues [4, 15, 19, 20]. The average number of drugs per encounter falls outside of the upper boundary of WHO standard which mandates the average value to be less than or equal to two (ideally, 1.6–1.8) in outpatient settings [4, 21].The average value was obtained low in HFSUH as it is a tertiary care teaching hospital of Haramaya University and hence limited number of drugs might be expected per prescription. The overall practice was not actually far from the WHO cut-off point and may be appreciated considering the context of developing countries like Ethiopia where healthcare delivery has become often erratic and empirical. The finding is also better than previous studies conducted in different settings within Ethiopia and abroad [18, 22–31]. However, lower average numbers of drugs per encounter (1.8, 1.9, 2.1, 1.9, 1.76 and 1.84) was reported from different settings [5, 6, 8, 10, 32, 33]. Two decades review report of 104 countries (in all WHO regions) indicated that average number of drugs prescribed per patient increased from 2.1 to 2.8 from 1900 to 2009 [34]. High average value can be associated with adverse consequences including drug-drug interactions, adverse drug reactions, and wastage of resources for the patient and healthcare system. Evidence based- and/or definitive therapy is mandatory to reduce the burden of drugs prescribed for the patient at a time [2].

Among the core prescribing indicators, WHO gives special emphasis for antimicrobials and injection prescribing practice as they have been mostly utilized but commonly misused class of drugs [4, 21]. In this study, the percentage of encounters with antimicrobial (s) and antibacterial (s) prescribed were found to be 66.9% (60.5% in HFSUH to 74.3% in SECIIIH) and 61.4% (56.5% in HFSUH to 65.2% in SECIIIH), respectively. This study revealed that the prevalence of antimicrobial containing prescription was more in police and military hospitals than the public hospitals (JH and HFSUH) had. These special government hospitals might have empirically prescribed antimicrobials without sufficient clinical evidence or the competency and experience of the prescribers might have also contributed for such difference. Apart from this, what makes all hospital in common is that there is no both culture and antimicrobial susceptibility (antibiogram) testing. Antimicrobial prescribing is solely based on clinical not microbiological evidence. Moreover, the highest number of antibacterial agents (per prescription) was observed in FHPH (1.18). For countries like Ethiopia where the infectious diseases are considered highly prevalent, prescribing antibiotics has become a prevailing practice. WHO set a maximum cut-off point on percent encounter of antibiotics in the outpatient settings of such countries (30%; ideal range, 20–26.8%) [4]. Our finding is more than twice the upper limit of WHO standard. Values that are lower than the present finding were reported from various healthcare settings including 30.3 and 24.27% in Indian tertiary-care hospitals [23, 31]**,** 52.8% in Dessie referral hospital [5], 28.1% in Nigerian army hospital [35], 52.% in Bahawal Victoria hospital, Pakistan [26], and 29.14% in Gondar University referral hospital [10]. In contrary, even more deviation were presented from several studies such as 70.6% in Bule Hora hospital, southern Ethiopia, [22] and 78% in tertiary-care hospital of Bangladesh [25]. Summoro et al. also reported that the percent encounters with antibiotics ranged from 46.7 to 85% in four hospitals of southern Ethiopia [9]. In broader sense, Holloway et al. reported the pecenatge of patients receiving antibiotics had increased from 45 to 54% in drug utilization review of 104 countries included from all WHO regions [34]. Empirical use of antimicrobial agents has come with emergence of AMR in several healthcare settings [4, 15]. If the current practice continues without any intervention, we are running to a post-antibiotic era where all the current antimicrobial agents will become historical. WHO (2014) report on global surveillance of AMR indicated that AMR is no longer a prediction for the future; it is an event happening right now, across the world, and is putting at risk the ability to treat common infections. Legitimate and responsible use of antimicrobials is, therefore, necessary to prevent selection of AMR and to minimize unnecessary wastage of scarce resource in developing countries [15, 19, 20, 36, 37]. It is imperative that antimicrobial stewardship programs should be established to preserve the existing antimicrobials and to contain AMR.

Concerning the injection practice, nearly one-fourth (26.5%) of prescribing encounters had at least one injectable with the highest (43.67%) and lowest (5.3%) values recorded in HFSUH and SECIIIH, respectively. In outpatient settings, WHO limits the prevalence of prescribing encounters with injectables to be less than 25% (ideal range, 13.4–24.1%) [4]. Injection prescribing practices of HFSUH (43.67%) and JH (36.5%) were too far from the upper limit of WHO standard. This might be related to the public nature of these hospitals (patients irrespective of their socio-demographic status could come to these settings unlike police and military hospitals) and the hence the injection demand was found higher. In addition, patient attendees who visited these hospitals might come with severe illness and/or complications to which injections were a primary choice. The overall injection practice is higher than previous studies conducted in Ethiopian healthcare settings and abroad [18, 22, 23, 27, 30, 31]. Comparable injection practices were also reported in different settings as 31% in Dilchora Referral Hospital (DRH) [5], 28.3% in selected hospitals of west Ethiopia [6], 38.1% in Hawassa university teaching and referral hospital [8], and 28.5% in Gondar university teaching and referral hospital [10]. In developing countries, up to 56% of primary care patients received injections. From this, over 90% injections deemed medically unnecessary. Globally, 15 billion injections were given to patients, but half of these injections were not sterilized, which might result in the transmission of potentially infectious diseases. Potential infections attributable to unsafe injection are hepatitis B (33%), hepatitis C (42%), and HIV (2%) [38, 39]. Frequent and impropriate use of injections might be attributable to psychological dependence of both patients and healthcare professionals. Despite the presence of safer, cheaper and more convenient oral alternatives, patients may preferably seek injections for treating their healthcare conditions assuming that injections are more effective than any other agents. Indeed, injections are important formulations in certain clinical conditions including emergency situations, and when other alternatives are not feasible. However, they are not without potential limitations. Frequent use of injections can result in physiological and psychological pain during injection; risk of transmission of potentially infectious biohazards and wastage of resources, among others [4].

In this study, the prevalence of drugs prescribed with generic name and from EDL of Ethiopia were 89.02% (86.53–94.02%) and 88.42% (77.56–94.02%), respectively. In this regard, the ideal WHO standard to which healthcare setting should achieve is 100% [4]. Even if the overall prescribing practice seems appreciable, there is a need to invest more efforts to meet the ideal targets of WHO. Being a tertiary care teaching hospital, HFSUH had a higher generic prescribing practice than the rest hospitals. In contrary to our findings, Prakash et al. reported that the percentage of drugs prescribed by generic name and from EDL were 0.5 and 53%, respectively [23]. There was also 0.0% generic prescribing practice as reported from tertiary hospital of Bangladesh [25]. This gap might be, in part, related to variation in the healthcare system, knowledge and experience of prescribers, healthcare policies and regulations (e.g. generic substitutions) and the sociodemographic indices of the countries. In concordant with the present finding, comparable generic prescribing practices were reported from different directions as 89.88% in tertiary hospital of rural India [31], 93.9% in DRH [5], and 90.61% in eastern Ethiopia [27]. Better than the present practice were also reported from previous findings [7, 8, 28, 40]. Being generic version is one of the criteria of essential drugs selection. In low-income countries like Ethiopia where resources are often scarce, prescribing with generic name has a multitude of advantages: generic drugs are relatively affordable, accessible, and recallable compared to brand counterparts [4]. It also reduces the likelihood of perverse financial incentives among healthcare workers of private and public healthcare settings.

Regarding the percentage of drugs prescribed from EDL, the overall finding did not meet the ideal target set by WHO (100%) [4]. Closely related findings were reported in previous studies [7, 8, 26, 28]. Prescribing from the EDL has several advantages. Essential drugs are the one that are selected by criteria including generic version, consideration of drugs of choice for the prevailing disease condition in the catchment area or country, cost-effectiveness ratio, quality, safety, risk-benefit aspect, and other pharmacokinetic considerations. Therefore, prescribing from EDL maximizes affordability and availability of drugs, reduces the possibility of drug interactions and adverse drug reactions and ultimately promotes patient therapeutic outcome [4, 38, 39]. To this end, WHO essential medicines policies are associated with improved quality use of medicines, particularly in low income countries. Low income countries reporting implementation of such policies have showed much better medicine use [41].

Concerning the healthcare professional’s information, the prevalence of dispenser name or signature was found less than 10%. This practice can vividly show the act of negligence and avoidance of accountability for drug related problems. This poor recording practice might be attributable to high patient load per dispenser and lack of strict rules and regulations governing the prescribing practice.

Looking at the distribution of drug classes and individual agents, antimicrobial drugs were the most frequently prescribed classes of drugs followed by analgesics and gastrointestinal agents. This result is in line with the high prevalence of antimicrobial containing prescriptions in the indicator study. Besides, amoxicillin and ciprofloxacin were the top two drugs prescribed under antimicrobials. In concordant with the present findings, the most common categories of drugs, reported by Pathak et al., were antibiotics (24.64%) followed by anti-diabetic drugs (12.38%), analgesics (12.23%), and cardiovascular agents (11.82%) [31]. Besides, the most commonly prescribed forms of antibiotics were amoxicillin (16.4%), ampicillin (15%), gentamicin (14.9%) and chloramphenicol (11 .6%) [8]. Sisay et al also reported that the most common antibiotic prescribed was amoxicillin followed by ciprofloxacin [27].

### Strength and limitation

This study tried to address several aspects of prescription beyond the usual indicator studies. A data abstraction format included general features of prescription and its completeness form as well as WHO prescribing indicator form to generate sufficient data. However, this study was not without potential limitations. It is a retrospective cross-sectional study, and hence some degree of documentation gap might be expected. This is also a quantitative descriptive study which could not answer the underlying causes why this problem exists. It simply highlighted the major problem areas for further action.

## Conclusion

Generally, JH and SECIIIH were not using standard prescriptions and the overall prevalence of standard prescriptions was less than 50%. Nearly one in ten prescription papers had a written clinical condition (diagnosis) with nil recording practice in SECIIIH. The tendency of prescribing drugs with dose and dosage form was very poor. Overall, none of the core prescribing indicators was in line with the WHO standard. These and other related problems should be investigated in-depth to find out the underlying problems. As drug use is highly vexed, a multitude of strategies (educational, economic, managerial and regulatory) should be designed to reverse the existing trends of drug use pattern (prescribing pattern in particular) in low and middle-income countries like Ethiopia. Evidence-based and/or definitive therapy reduces the burden of antimicrobial drugs prescription thereby contains the emergence and spread of AMR; limits adverse drug reactions, and contraindications, and possibly avoids unnecessary direct and indirect healthcare costs.

## Abbreviations

AMR: Antimicrobial Resistance
DRH: Dilchora Referral Hospital
EDL: Essential Drug List
FHPH: Federal Harar Police Hospital
FMHACA: Food Medicine and Healthcare Administration and Control Authority
HFSUH: Hiwot Fana Specialized University Hospital
JH: Jugel Hospital
SECIIIH: Southeast Command III Hospital
WHO: World Health Organization
